# Additive Manufacturing of Textured FePrCuB Permanent Magnets

**DOI:** 10.3390/mi12091056

**Published:** 2021-08-31

**Authors:** Dagmar Goll, Felix Trauter, Ralf Loeffler, Thomas Gross, Gerhard Schneider

**Affiliations:** Materials Research Institute, Aalen University, 73430 Aalen, Germany; felix.trauter@hs-aalen.de (F.T.); ralf.loeffler@hs-aalen.de (R.L.); thomas.gross@hs-aalen.de (T.G.); gerhard.schneider@hs-aalen.de (G.S.)

**Keywords:** additive manufacturing, PrFeCuB, selective laser melting (SLM), laser powder bed fusion (L-PBF), coercivity, book-mold-cast magnets, permanent magnets, tailored microstructure

## Abstract

Permanent magnets based on FePrCuB were realized on a laboratory scale through additive manufacturing (laser powder bed fusion, L-PBF) and book mold casting (reference). A well-adjusted two-stage heat treatment of the as-cast/as-printed FePrCuB alloys produces hard magnetic properties without the need for subsequent powder metallurgical processing. This resulted in a coercivity of 0.67 T, remanence of 0.67 T and maximum energy density of 69.8 kJ/m^3^ for the printed parts. While the annealed book-mold-cast FePrCuB alloys are easy-plane permanent magnets (BMC magnet), the printed magnets are characterized by a distinct, predominantly directional microstructure that originated from the AM process and was further refined during heat treatment. Due to the higher degree of texturing, the L-PBF magnet has a 26% higher remanence compared to the identically annealed BMC magnet of the same composition.

## 1. Introduction

Additive manufacturing based on laser powder bed fusion L-PBF promises a variety of new opportunities for functional materials: customized material properties, new component and topology structures, and individual components of complex geometry and functional integration. For permanent magnets, as important key materials for electrification, new degrees of freedom in the development and design of products may also be expected. For example, L-PBF has potential for texture/grain orientation control and specifically tailored microstructures. However, L-PBF technology is challenging, especially for today’s strongest permanent magnets based on rare earth (RE) metals such as Fe-Nd-B. The three main challenges are: First, RE-based material is highly sensitive to oxidation. Second, there is a lack of powders with spherical morphology and suitable chemical compositions. Third, good permanent magnet properties require specific microstructures. Such favorable microstructures are usually composed of small hard magnetic grains on the micron or nanometer scale, magnetically isolated by a nonmagnetic grain boundary phase. To achieve such microstructures, a powder metallurgical process (sintering) or rapid quenching is traditionally required. Since the L-PBF process produces cast-like structures, this initially represents a contradiction. Nevertheless, it was recently shown for Fe-Nd-B, that rapid solidification in L-PBF can be realized. Precisely tuned processing parameters result in shallow melt pools, leading to nanocrystalline microstructures in the bulk showing hard magnetic properties [1,2,3,4,5]. One strategy is trying to realize microstructures by L-PBF processing that were previously only possible using powder metallurgy or rapid quenching. Alternatively, nanocrystalline powder such as MQP-S powder from Magnequench can be processed. When during laser processing the gas atomized spherical powder particles are melted by the laser exclusively at their surfaces, the original isotropic nanostructure can be preserved [6,7]. As the composition of MQP-S prohibits the formation of an RE-rich grain boundary phase (and therefore the formation of larger coercivities), intermixing of low-melting eutectic powder before printing [7] or infiltration of low-melting eutectic powders afterwards [6] may result in coercivity enhancement.

Another strategy is to choose magnet materials for L-PBF processing where permanent magnet properties can be realized in the as-cast state (e.g., by annealing) without the need for subsequent powder metallurgical processing or rapid quenching. Such magnet materials are AlNiCo, CoSm (17:2) and FePrCuB. In AlNiCo (shape anisotropy magnet), spinodal segregation results in single-domain FeCo needles in a paramagnetic AlNi matrix [8]. White et al. [9,10] realized such magnets through additive manufacturing. The magnets showed somewhat better properties than cast magnets (coercivity *µ*_0_*H*_c_ = 0.18 T, remanence *J*_r_ = 0.9 T, maximum energy density (*BH*)_max_ = 47.7 kJ/m^3^) due to finer microstructures and columnar grain growth. In (CoCuFeZr)_17_Sm_2_ (pinning-hardened magnet) a nanoscale precipitation structure of different hard magnetic phases forms in a self-organized process during a three-step annealing procedure (homogenization, isothermal heat treatment, slow cooling) [11,12]. Goll et al. demonstrated that additive manufacturing of such magnets is also possible including texture in the printed parts (*µ*_0_*H*_c_ = 2.77 T, *J*_r_ = 0.78 T, (*BH*)_max_ = 109.4 kJ/m^3^) [13].

In FePrCuB (nucleation-hardened magnet), primary solidified regions of hard magnetic Fe_14_Pr_2_B (14:2:1) in the micron range and paramagnetic Pr-rich and PrCu-rich phases are already present in the as-cast state [14,15,16,17]. Furthermore, a soft magnetic Fe phase occurs [14,15,16,17]. A subsequent two-step heat treatment is required to develop large coercivities [14,16,17]. Step 1 is a homogenization treatment at 1000 °C to eliminate the soft magnetic Fe phase. During homogenization, Fe_17_Pr_2_ is formed and the primary Pr-rich and PrCu-rich phases form a Pr/PrCu-rich eutectic phase. Thus, after homogenization treatment the phases 14:2:1, 17:2, Pr/PrCu eutectic and remnants of the original Pr-rich type are present. Step 2 is an annealing treatment at temperatures of around 500 °C similar to the post-annealing of Fe-Nd-B sintered magnets. During Step 2 most of the 17:2 phase is eliminated by forming Fe_13_Pr_6_Cu_1_. Therefore, after the second annealing step the following phases occur: 14:2:1, 13:6:1, Pr/PrCu eutectic and Pr-rich as well as remnants of 17:2. Quenching is not required for either heat treatment step. Cooling in the furnace leads to even larger coercivities. Boron content has to be between 3.5 and 4.5 at% to avoid the formation of the Fe_4_Pr_1.1_B_4_ (η-phase) [14,15,16,17,18]. It is thus about half that of alloys used for Fe-Nd-B sintered magnets. Furthermore, the amount of boron significantly influences the shape and size of the hard magnetic grains. For 3.7 at% platelets of size (2–5 µm) × (10–20 µm) were observed, whereas for 4.5 at% rather spherical grains of size 10–20 µm do occur [17]. The Pr content primarily influences the hard magnetic properties. The highest coercivities were obtained for a Pr content as large as 20 at% [17]. The addition of 1–2 at% Cu also favors the formation of larger coercive fields [19]. On the one hand, this may be due to the formation of low melting Pr/PrCu eutectic, which improves grain boundary wetting and magnetic isolation of adjacent grains [16,17,20]. On the other hand, the amount of soft magnetic Fe_17_Pr_2_ (Curie temperature found around room temperature, 10–37 °C [21,22,23]) can be mostly eliminated by the formation of Cu containing Fe_13_Pr_6_Cu_1_ [16,17]. Fe_13_Pr_6_Cu_1_ acts as an additional magnetic isolating grain boundary phase. It further appears that Fe_13_Pr_6_Cu_1_ better isolates the remaining soft magnetic Fe_17_Pr_2_ phase [21]. Due to the comparatively low Curie temperature of Fe_17_Pr_2_ and the embedding into the Fe_13_Pr_6_Cu_1_ matrix, the influence of the Fe_17_Pr_2_ phase on the magnetic properties is negligible at room temperature [21]. It was found by Mössbauer spectroscopy and magnetometry that Fe_13_Pr_6_Cu_1_ is antiferromagnetic with a Néel temperature of 391 K [24]. Finally, due to the additive Cu the duration of both annealing steps can be significantly reduced. Under certain conditions, e.g., faster cooling at the mold wall, directional solidification (magnetic texture) and thus larger remanence is observed [16,17,25]. The best magnetic properties reported so far for as-cast material were obtained for the chemical composition Fe73.8-Pr20.5-Cu2.0-B3.7 and two-step heat treatment (step 1: 1000 °C/5 h; step 2: 500 °C/3 h) yielding *J*_r_ = 0.62 T, *µ*_0_*H*_c_ = 1.13 T and (*BH*)_max_ = 70.0 kJ/m^3^ [17]. Recently, limited trials of laser melting for a few powder layers of a similar composition (Fe73.5-Pr21.0-Cu2.0-B3.5) resulted in very small samples showing a remanence of *J*_r_ = 38 Am/kg and a coercive field of *µ*_0_*H*_c_ = 0.75 T [26].

In this paper we demonstrate that significantly larger bodies of FePrCuB can be realized by additive manufacturing. After suitable heat treatment, they exhibit permanent magnet properties that are comparable to annealed conventionally cast FePrCuB material of the same composition without the necessity for subsequent powder metallurgical processing. This requires powder of a suitable composition and a special inert gas process chamber (including suitable laser processing parameters and parameters for two-step post-annealing). The feasibility study is performed in direct comparison with conventionally fabricated as-cast and heat-treated FePrCuB. Besides characterization of the microstructure and magnetic properties of the as-built/as-cast states and the annealed states, the potential of texture formation in the 3D-printed components is shown and evaluated.

## 2. Experimental Procedures

**Figure 1:**
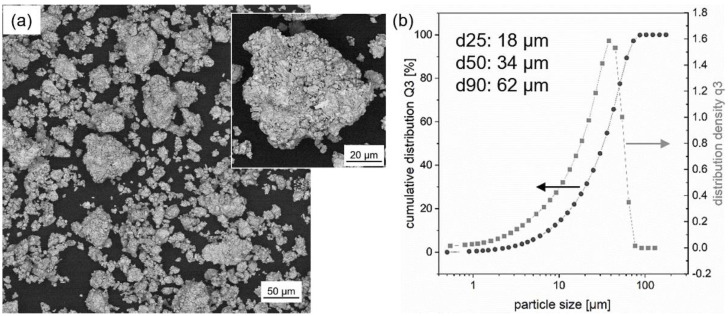
Fe73.8-Pr20.5-Cu2.0-B3.7 powder: (**a**) Powder used in the L-PBF process (scanning electron microscopy image, backscatter electron detector). A higher magnification image of the powder is shown as inset of (**a**). (**b**) Particle size distribution (cumulative distribution *Q3*, distribution density *q3*) of the powder using laser diffraction. The values of *d25*, *d50* and *d90* are also listed.

For the experiments, the alloy composition Fe73.8-Pr20.5-Cu2.0-B3.7 (at%) was chosen for which the best hard magnetic properties were reported in literature so far [16,17]. First, book-mold-cast (BMC) Fe73.8-Pr20.5-Cu2.0-B3.7 material was fabricated by induction melting (VTC 200 V/Ti, Indutherm, Walzbachtal, German) in Ar atmosphere from the constituent elements (purity > 99.9%) and a Fe-B pre-alloy. Next, the produced BMC material was mechanically pre-shredded and subsequently ball-milled under Ar atmosphere (O_2_ < 1 ppm). Finally, the resulting powder was mechanically sieved (63 µm sized sieve, 230 mesh size). For additive manufacturing the powder fraction <63 µm was chosen (Figure 1a). It was further characterized by laser diffraction (HELOS BR, Sympatec, Clausthal-Zellerfeld, Germany). Analysis was conducted using a lens system with a measuring range of 0.5–175 µm. (Figure 1b). The measured particle distribution showed a distinct peak at around 46 µm particle size. The *d25*, *d50* and *d90* values were found to be 18 µm, 34 µm and 62 µm, respectively. Despite the non-spherical shape of the powder particles, the coating worked well in the process chamber and a high powder density was achieved in the powder bed. Lab scale L-PBF of the samples was conducted in a specially developed process chamber that can be loaded and operated inside a glovebox system [27]. The chamber allows processing of very small quantities (<350 mm^3^) of sensitive powders under very pure Ar atmosphere (O_2_ < 20 ppm). To perform the L-PBF experiments the chamber was connected to a fiber laser (TruFiber 1000, TRUMPF, Ditzingen, Germany) with a maximum output power of 1000 W and laser wave length of 1070 nm. The size of the printed cuboids built on a steel substrate plate was 4 mm × 4 mm × 2 mm. Processing parameters were laser spot diameter 46 µm, hatch distance 46 µm, layer thickness 100 µm and laser power 200 W. The laser scanning speed was varied between 200 and 2.000 mm/s. The scanning strategy was realized in parallel lines with alternating direction (forward–backward). Selected as-printed samples were annealed under Ar atmosphere according to the following two-step procedure: (1) homogenization (temperature *T* = 1000 °C, duration *t* = 5 h; slow cooling) and (2) aging (*T* = 500 °C, *t* = 3 h, slow cooling). The original BMC material is used as a benchmark (reference). It was subjected to the same two-step annealing procedure as the printed samples. Table 1 gives an overview of the samples investigated in this work.

The macroscopic magnetic properties (coercivity *H*_c_, remanence *J*_r_, maximum energy density (*BH*)_max_) of the BMC magnets and L-PBF magnets were determined from hysteresis loop measurements (PPMS-9T, QuantumDesign, Darmstadt, Germany) at room temperature. For converting the remanence in Tesla, a material density of 7.4 g/cm^3^ was used. For microstructure analysis, polished microsections of the samples were produced using metallographic techniques. The microstructure was characterized in an optical microscope (Axio Imager.Z2m, ZEISS, Jena, Germany, bright field and polarized light) and in a scanning electron microscope (Sigma 300 VP, ZEISS, Jena, Germany). Scanning electron microscopy including energy dispersive X-ray analysis (EDX) was used to determine both the chemical composition of the magnet samples produced and the phases occurring in them. Grain orientation and texture were investigated using scanning electron microscope (SEM) and electron backscatter diffraction (EBSD). EBSD data was collected with a Hikari camera (EDAX-Ametek, Weiterstadt, Germany) and statistically analyzed with OIM v8.6 (orientation imaging microscopy) software. Furthermore, X-ray diffraction (XRD) analysis (XRD 3003, CoK_α_ radiation, Bragg Brentano geometry, GE Seifert, Schnaittach-Hormersdorf, Germany) was carried out in order to validate the phases deduced from EDX analysis.

## 3. Analysis of L-PBF Printed Parts

### 3.1. Selection of Suitable Processing Parameters

**Figure 2:**
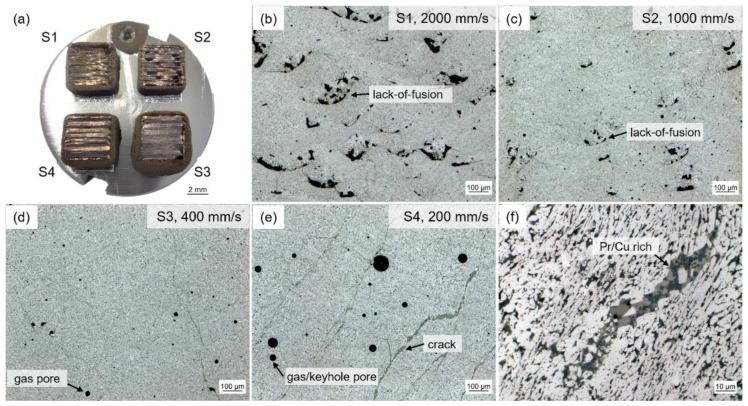
Printed parts of the Fe73.8-Pr20.5-Cu2.0-B3.7 powder. (**a**) Macrograph of four different laser scanning speeds (S1: 2000 mm/s (VED: 21.7 J/mm^3^), S2: 1000 mm/s (43.5 J/mm^3^), S3: 400 mm/s (108.7 J/mm^3^), S4: 200 mm/s (217.4 J/mm^3^)) in top view. (**b**–**e**) Optical microscopy images of corresponding microstructures of the four samples S1 (**b**), S2 (**c**), S3 (**d**) and S4 (**e**). (**f**) Pronounced crack in sample S4 filled with eutectic phase. Relative densities of the samples are for S1: 93%, S2: 94%, S3: 97% and S4: 93%.

For the selection of suitable processing parameters, four different laser scanning speeds—2000 mm/s, 1000 mm/s, 400 mm/s and 200 mm/s—were chosen. They are related to volume energy densities (VED) of 21.7 J/mm^3^, 43.5 J/mm^3^, 108.7 J/mm^3^ and 217.4 J/mm^3^, respectively. This covers a large possible process window typically used for either realizing fine, textured or both structures [4,27]. Figure 2 shows the printed parts (Figure 2a) and corresponding microstructures (Figure 2b–e). A relief of the scan tracks is visible on the surfaces of the printed parts. With increasing VED an increasing amount of loosely sintered powder adheres to the lateral faces of the printed parts. For 400 mm/s (Figure 2d) the number of defects is smallest and the relative density is highest (97%). For larger laser scanning speeds, the porosity increases significantly (Figure 2b,c) due to lack of fusion. This indicates an insufficient energy input to fully melt the powder. For smaller laser scanning speeds, pronounced cracks and gas pores or keyhole pores occur due to excessive energy input (Figure 2e). The latter printed part shows the smoothest sample surface, but also the largest amount of adhering powder particles. This indicates a strong heat effect on the surrounding powder from the high energy input. It has to be noted that cracks formed at an elevated temperature are filled directly with the liquid phase, i.e., Pr/Cu rich eutectic (Figure 2f). Regarding the microstructure and density of the printed parts, a laser scanning speed of 400 mm/s (VED of 108.7 J/mm^3^) offers the best starting point for further parameter optimizations. Therefore, this parameter set was selected for further in-depth investigations in this work.

### 3.2. Evolution of Magnetic Properties

**Figure 3:**
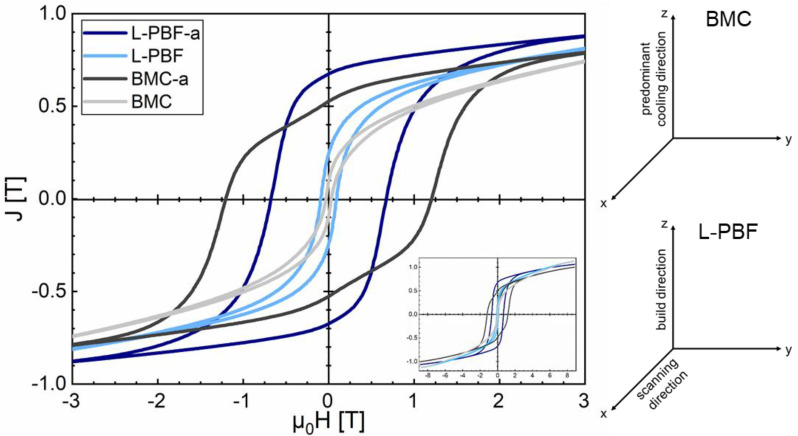
Room temperature hysteresis loops of 3D-printed Fe73.8-Pr20.5-Cu2.0-B3.7 in the as-built state (*L-PBF*) (light blue) and annealed state (*L-PBF-a*) (blue) and the reference BMC material in the as-cast state (*BMC*) (light gray) and annealed state (*BMC-a*) (gray) in comparison. The magnetic field was applied along *x* direction (L-PBF: laser scanning direction, BCM: parallel to mold wall).

**Figure 4:**
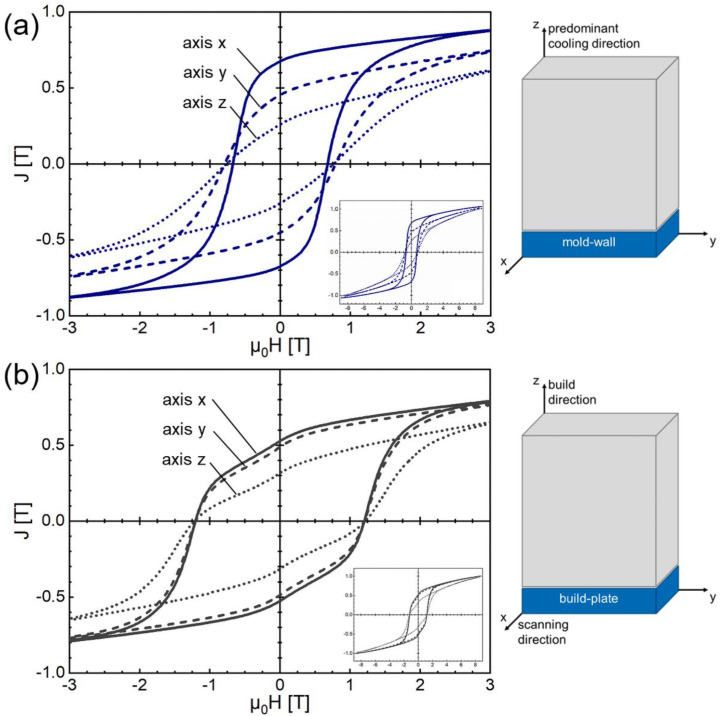
Room temperature hysteresis loops of (**a**) 3D-printed Fe73.8-Pr20.5-Cu2.0-B3.7 in the annealed state (*L-PBF-a*) and (**b**) BMC Fe73.8-Pr20.5-Cu2.0-B3.7 in the annealed state (*BMC-a*). The external magnetic field has been applied in three different directions (Cartesian coordinate system) as illustrated in the inset.

In Figure 3 the room temperature hysteresis loops of additively manufactured FePrCuB in the as-built state (*L-PBF*) and annealed state (*L-PBF-a*) are represented in comparison with the hysteresis loops of the reference BMC material in the as-cast state (*BMC*) and annealed state (*BMC-a*). As expected, in the as-built/as-cast state the samples show no hard magnetic properties (*L-PBF*: *µ*_0_*H*_c_ = 0.09 T, *J*_r_ = 0.25 T, (*BH*)_max_ = 4.0 kJ/m^3^; *BMC*: *µ*_0_*H*_c_ = 0.03 T, *J*_r_ = 0.1 T, (*BH*)_max_ = 0.4 kJ/m^3^). The two-step annealing procedure is essential for the development of the hard magnetic properties. In the annealed state the printed part (*L-PBF-a*) shows good hard magnetic properties. A coercivity of *µ*_0_*H*_c_ = 0.67 T, remanence of *J*_r_ = 0.67 T and maximum energy density of (*BH*)_max_ = 69.8 kJ/m^3^ were measured. In comparison, the annealed reference BMC material (*BMC-a*) yields *µ*_0_*H*_c_ = 1.20 T, *J*_r_ = 0.53 T and (*BH*)_max_ = 42.9 kJ/m^3^. It becomes evident that the reference BMC magnet has a higher coercivity than its additively manufactured counterpart. However, the printed specimen shows a larger remanence (and therefore maximum energy density) compared to the reference BMC magnet (26% increase). For the hysteresis measurements the magnetic field was applied along the *x* direction (L-PBF: laser scanning direction, BCM: parallel to mold wall). To investigate whether there are texture effects, further hysteresis measurements were performed with the magnetic field applied along the *y* direction (L-PBF: perpendicular laser scanning direction, BCM: parallel to mold wall but perpendicular to *x*) and along the *z* direction (L-PBF: parallel to build direction, BCM: perpendicular to mold wall). In Figure 4 the hysteresis loops of all three directions are shown for *L-PBF-a* (Figure 4a) and *BMC-a* (Figure 4b). The magnetic properties *µ*_0_*H*_c_, *J*_r_ and (*BH*)_max_ of all hysteresis loop measurements are summarized in Table 2. It clearly follows that in both cases a magnetic texture is present. However, this effect is much more pronounced in the *L-PBF-a* specimen than in the *BMC-a* specimen. For *L-PBF-a* the magnetic texture is most pronounced along the laser scanning direction (*x* direction). Therefore, the *x* direction can be regarded as an easy axis for preferred magnetization orientation. In contrast, for *BMC-a* the mold wall acts as an easy plane for preferred magnetization orientation.

### 3.3. Evolution of Microstructure

**Figure 5:**
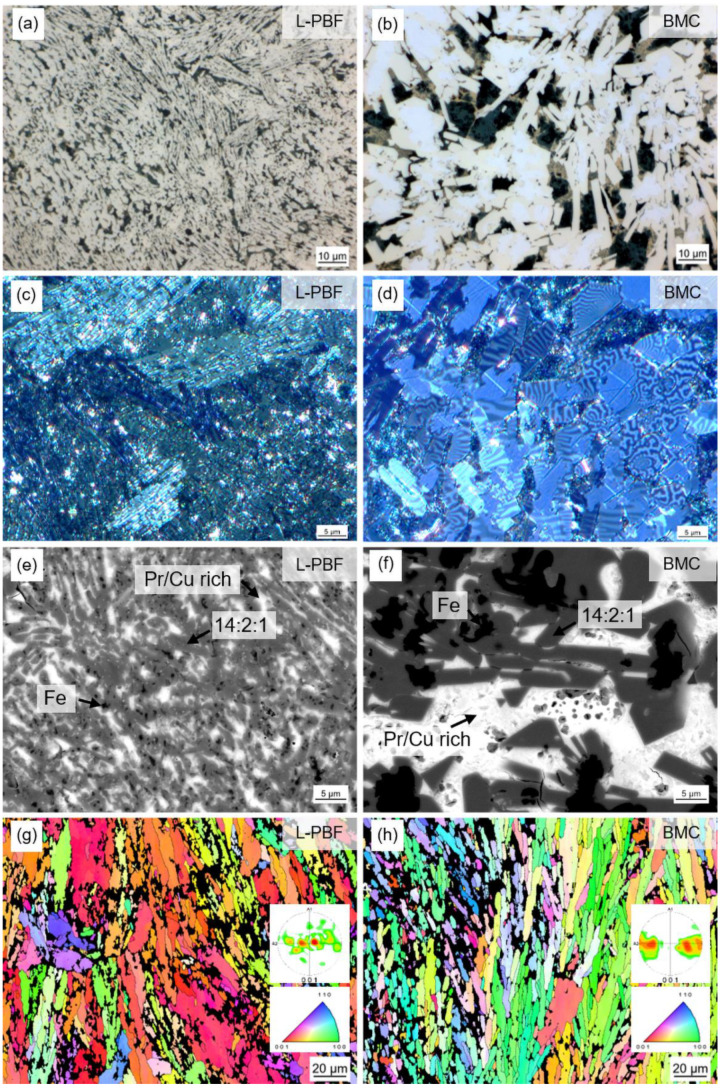
Microstructure of 3D-printed (L-PBF) Fe73.8-Pr20.5-Cu2.0-B3.7 observed for polished microsections in the as-built state (*L-PBF*) (left) in comparison with BMC material of the same composition (*BMC*) (right): (**a**,**b**) Optical microscopy image. (**c**,**d**) Kerr microscopy image. (**e**,**f**) SEM image (backscatter electron detector). (**g**,**h**) EBSD analysis (inverse pole figure IPF). *L-PBF* shows the same phase composition as *BMC* but with finer grain structure. Both samples show partially textured microstructures: in the printed specimen, the *c* axes generally point out of the image plane, while in the cast specimen, the *c* axes are oriented parallel to the mold wall but do not take any other preferred orientation in this plane. Microsections were made in the *z–y* plane (same definition of axes in Figure 4).

In Figure 5 the microstructure of additively manufactured FePrCuB in the as-built state is shown (*L-PBF*) in comparison with BMC material as a reference (*BMC*). The samples were investigated using optical microscopy in bright field (Figure 5a,b) and Kerr mode (Figure 5c,d) as well as scanning electron microscopy in backscattered electron mode coupled with EDX (Figure 5e,f) and EBSD mode (Figure 5g,h). It is obvious from Figure 5a–f that L-PBF of FePrCuB powders results in much finer primary Fe_14_Pr_2_B crystals compared to BMC material. The as-printed structure resembles the fine microstructure with a finely dispersed Nd-rich phase observed in additively manufactured ternary Fe75-Nd18-B7 [28]. The phases occurring in both FePrCuB sample types were deduced from the chemical composition determined by EDX analysis. Based on the measured compositions, the following phases are identified: Fe_14_Pr_2_B, Fe as well as Pr-rich and PrCu-rich (Table 3).

The composition of the individual phases in sample *L-PBF* and sample *BMC* as well as their total compositions are comparable. As boron cannot be detected via EDX analysis, the theoretical compositions of the phases were first calculated without boron and then compared to the measured ones. The pole figures plotted from the EBSD measurements confirm partially textured microstructures for both samples. In the case of the *L-PBF* sample, grains are generally aligned with their *c* axis perpendicular to the build direction and parallel to the laser scan direction. The preferred direction of magnetization thus points out of the imaged plane (out-of-plane). Because of the very fine grain structure of the *L-PBF* sample, neighboring lamellae of similar orientation are not always resolved as individual entities in the EBSD map but rather are recognized as a single grain. The pole figure of the *BMC* material shows that the *c* axes of the grains are aligned parallel to the mold wall and perpendicular to the predominant solidification direction with the highest cooling rate. Although there is a gap in the (001) direction (out-of-plane orientation), the band-like pattern suggests that the *c* axes are distributed randomly in the plane perpendicular to the predominant solidification direction.

**Figure 6:**
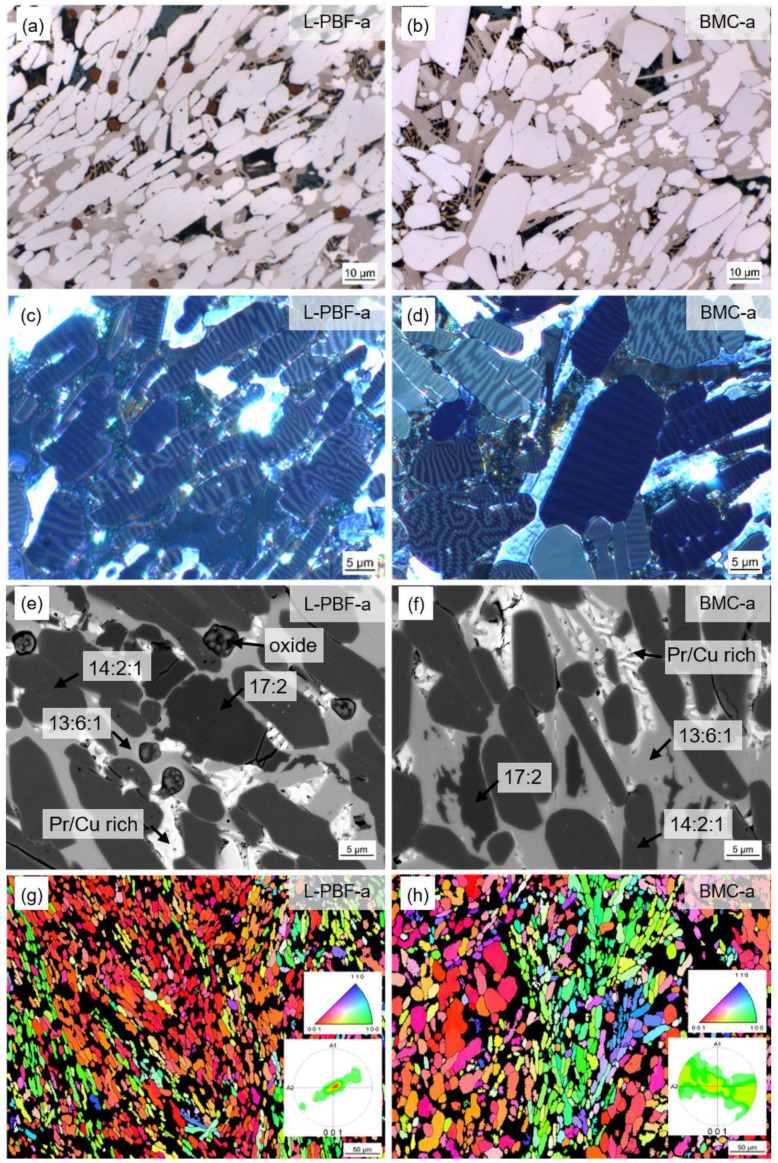
Microstructure of 3D-printed (L-PBF) and annealed Fe73.8-Pr20.5-Cu2.0-B3.7 observed for polished microsections in the as-built state (*L-PBF-a*) (left) in comparison with BMC material of the same composition (*BMC-a*) (right): (**a**,**b**) Optical microscopy image. (**c**,**d**) Kerr microscopy image. (**e**,**f**) SEM image (backscatter electron detector). (**g**,**h**) EBSD analysis (inverse pole figure IPF). *L-PBF-a* and *BMC-a* show the same phase composition and morphology. Original grain orientation patterns are preserved during heat treatment; however, the typical textures of both samples are more pronounced. Microsections were made in the *z–y* plane (same definition of axes in Figure 4).

During the two-step annealing procedure (for details, see Section 1—Introduction and Section 2—Experimental) the microstructure changes (Figure 6). In both samples the hard magnetic Fe_14_Pr_2_B grains grow and adopt a polygonal shape (Figure 6a,b). Together with Fe_17_Pr_2_ grains they are embedded in a grain boundary matrix. The grain boundary matrix is composed of Fe_13_Pr_6_Cu_1_ as well as Pr-rich and Pr/PrCu eutectic phases (Figure 6e,f and Table 3). Formerly present α-Fe is dissolved during heat treatment and is therefore not detected in the annealed state. The printed sample *L-PBF-a* contains additional RE oxides that are locally observed. The volume fractions of the different phases are quantified in Section 4. EBSD analysis shows that the original grain orientation patterns of the as-printed and as-cast state are preserved during heat treatment (Figure 6g,h). In the case of sample *L-PBF-a*, the pole figure shows an even more predominant out-of-plane orientation of *c* axes. Grains with in-plane orientation show a rotation of the *c* axis perpendicular to the primary solidification direction, similar to the behavior in the BMC sample. The primary solidification direction in this case is along the build direction (vertical in the image) with a slightly diagonal aspect perpendicular to the laser scanning direction. The observation of similarly oriented grains is also supported by Kerr images where individual grains show similar domain patterns (Figure 6c). The pole figure of sample *BMC-a*, on the other hand, now shows a completely closed band-shaped distribution pattern. The random distribution of the *c* axes in the plane perpendicular to the predominant solidification direction is also supported by the wide variety of domain patterns observed in the Kerr image (Figure 6d). For *BMC-a* the EBSD map shows that grains with out-of-plane orientation are generally larger than in-plane-oriented grains. This demonstrates the typical platelet structure of the grains, with the *c* axis oriented perpendicular to the preferred growth direction. For *L-PBF-a* this behavior is not as obvious, suggesting an overall smaller grain size. Average grain sizes were determined from EBSD analysis. The mean grain size for *L-PBF-a* and *BMC-a* amounts to about 12 µm and 17 µm, respectively.

**Figure 7:**
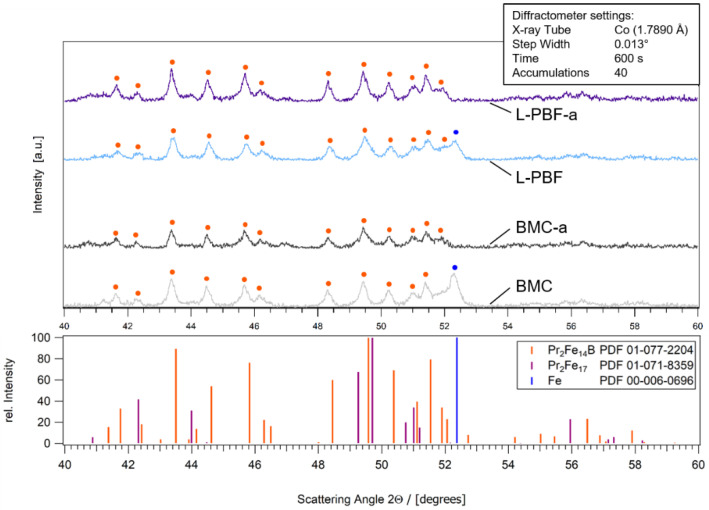
Diffractograms of 3D-printed Fe73.8-Pr20.5-Cu2.0-B3.7 in the as-built state (*L-PBF*) (light blue) and annealed state (*L-PBF-a*) (blue) and the reference BMC material in the as-cast state (*BMC*) (light gray) and annealed state (*BMC-a*) (gray) in comparison. As reference, the relative intensities of Fe_14_Pr_2_B, Fe_17_Pr_2_ and Fe from literature are shown. The phases that have been identified in the X-ray diffraction (XRD) chart are marked in the diffractograms.

The XRD diffractograms of samples *L-PBF*/*BMC* and *L-PBF-a*/*BMC-a* show very good agreement with respect to position and intensity of the measured peaks (Figure 7). This indicates that additively manufactured and conventionally cast FePrCuB contain similar phases of comparable composition in both the as-built/as-cast and annealed states. In all samples, the hard magnetic ϕ phase (14:2:1) was identified as the main phase in the diffractogram. In the as-cast (BMC) and as-built (L-PBF) sample, α-Fe was additionally identified. XRD analysis confirms that additive manufacturing results in similar structures as observed in conventional BMC material.

## 4. Discussion

### 4.1. Comparison between Fe-Pr-Cu-B-Based Printed Parts and BMC Material

The magnetic properties obtained for annealed BMC material are in good agreement with the magnetic properties obtained in the literature for cast material [16,17]. The brief trial of laser melting a few powder layers of Fe73.5-Pr21.0-Cu2.0-B3.5 as reported in [26] resulted in a coercive field of 0.75 T and remanence of 38 Am/kg. Assuming a density of 7.4 g/cm^3^, the remanence value is equivalent to 0.35 T.

**Figure 8:**
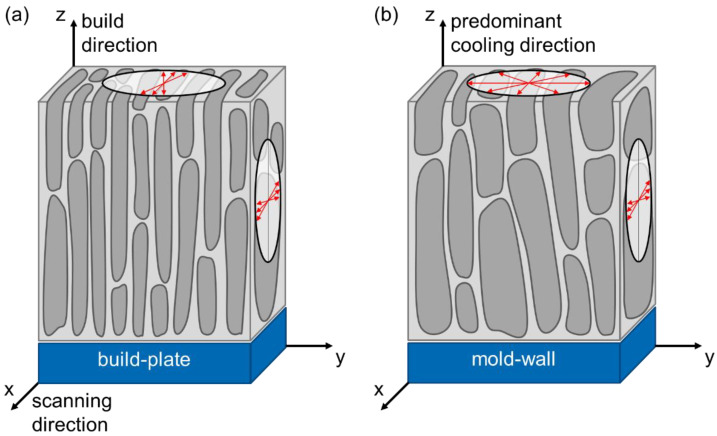
Schematic presentation of the FePrCuB sample orientation and allocation of the axes in the samples (**a**) *L-PBF-a* and (**b**) *BMC-a*. In both cases, the *z* axis is assigned to the direction with the highest temperature gradient during solidification. Based on results from Section 3, typical orientations of *c* axes of Fe_14_Pr_2_B grains are qualitatively indicated by red arrows.

Comparing the annealed BMC material (*BMC-a*) with annealed additively manufactured components (*L-PBF-a*) the coercivity (remanence) for *L-PBF-a* is 45% (26%) smaller (larger) than for *BMC-a*. From the investigations above it can be concluded that the printed magnets (L-PBF magnet) are characterized by a distinct, predominantly directional microstructure. It originates from the high temperature gradients during the AM process. In contrast, the annealed book-mold-cast FePrCuB sample shows planar isotropic magnetic behavior. The observation of this characteristic is consistent with data previously reported in literature [16,17,25]. Figure 8 shows a schematic representation of the FePrCuB samples and their orientation to the mold wall (*BMC*) and build plate (*L-PBF*), respectively. For *BMC*, the axis perpendicular to the mold wall has the highest cooling rate (*z* axis). The *y* axis and *x* axis are assumed to have similar temperature gradients and are thus interchangeable. In the case of *L-PBF*, the axis in the build direction has the highest cooling rate (*z* axis). The axis perpendicular to the scanning direction has an intermediate temperature gradient (*y* axis) whereas the axis along the scanning direction has the smallest temperature gradient (*x* axis). In Section 3 it is shown that the different cooling rates influence orientation of the *c* axis of the hard magnetic Fe_14_Pr_2_B grains and thus magnetic texturing.

**Figure 9:**
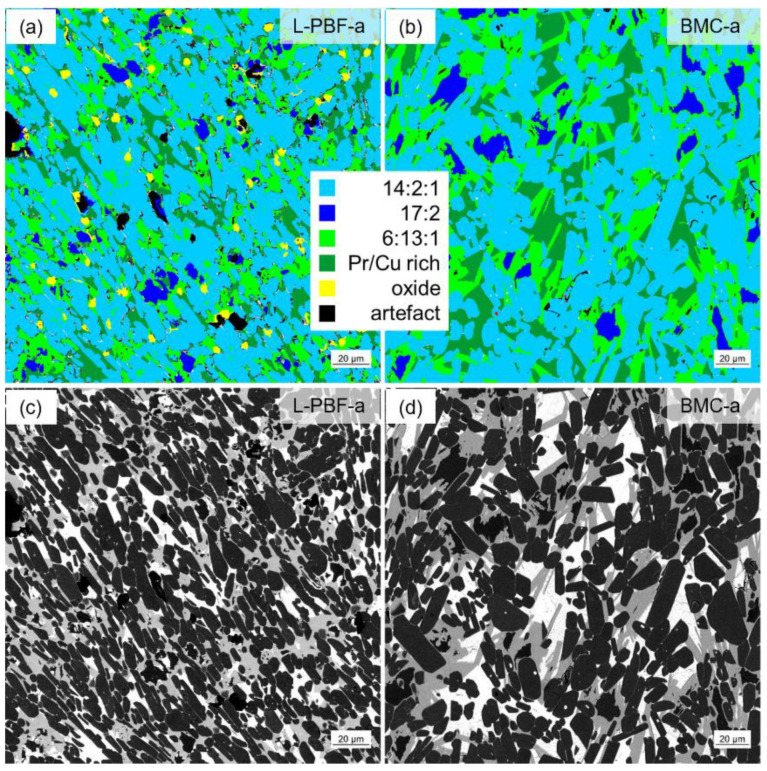
Quantitative image analysis for *L-PBF-a* in comparison with *BCM-a*: (**a**) Quantitative image analysis *L-PBF-a* (false color representation). (**b**) Quantitative image analysis *BMC-a* (false color representation). (**c**) SEM image used for quantitative image analysis *L-PBF-a*. (**d**) SEM image used for quantitative image analysis *BMC-a*. Both contain similar volume fractions of the 14:2:1 phase, Pr-rich phase and 17:2 phase. *BMC-a* contains slightly larger amounts of the 13:6:1 phase. *L-PBF-a* contains an additional 3% RE oxides.

Anisotropic magnets, in principle, show smaller coercivities compared to isotropic ones [29]. The nature of the grain boundary phase further influences coercivity, which will be considered in the following in more detail. In Figure 9, quantitative image analysis has been performed on SEM-BSE images for the *BMC-a* and *L-PBF-a* material. Images with a resolution of 110 nm/px at more than 10 randomly chosen positions within a sample area of 2 mm × 2 mm were acquired and subsequently processed with ZEN core image analysis software (ZEN core 3.1, Carl Zeiss, Oberkochen, Germany). The phases occurring in the images were separated by machine-learning-assisted threshold segmentation and the corresponding volume fractions quantified. From this analysis it turns out that both samples contain comparable volume fractions of the hard magnetic 14:2:1 phase (61 ± 1%), Pr-rich phase (12.5 ± 1.5%) and 17:2 phase (6 ± 1%). The amount of the 13:6:1 phase is slightly larger for *BMC-a* (approx. 21%) compared to *L-PBF-a* (approx. 17%). In the case of *L-PBF-a* material a small amount of RE oxides is detected (approx. 3%). Besides the isotropic character of *BMC-a*, the larger amount of magnetically isolating 13:6:1 and the absence of wetting-restricting oxides may be further reasons for the larger coercivity of *BMC-a* compared to *L-PBF-a*. Another influence on the coercivity is the grain size. For nucleation-hardened magnet materials it is well-known, that the coercivity increases with decreasing grain size [30]. As the relation between coercivity and grain size is logarithmic, the influence is significantly larger for smaller grain sizes. Regarding the grain sizes of 12 µm and 17 µm reported in Section 3.3 for *L-PBF-a* and *BMC-a*, respectively, this corresponds to a coercivity of about 0.15 T higher for *L-PBF-a*. However, this slight influence of the grain size on the coercivity is superimposed by the opposing effects mentioned above. The influence of soft magnetic 17:2 and antiferromagnetic 13:6:1 on the magnetic saturation polarization shall be negligible.

### 4.2. Evaluation of Texturing Effect

Using quantitative microstructural analysis in combination with magnetometry the degree of texturing can be roughly estimated. Assuming a magnetic saturation polarization of single-phase Fe_14_Pr_2_B of *J*_s_ = 1.56 T at room temperature [23] and simple diluting mechanisms, the saturation polarization of the analyzed sample containing approximately 62% hard magnetic 14:2:1 phase would amount to approximately 1 T. This is of the same order as the saturation polarization estimated by magnetometry from the approach to ferromagnetic saturation (*J*_s_ vs. 1/(*µ*_0_*H*)^2^ plot, *J*_s_ ≈ 1.1 T). In the case of ideal isotropy of the hard magnetic grains, the remanence amounts to about half of the saturation polarization, i.e., about *J*_r_ = 0.5 T. Related to this, a remanence of 0.67 T as observed for *L-PBF-a* corresponds to an enhancement of about 34% and to a texture grade of *J*_r_/*J*_s_ ≈ 0.67.

## 5. Conclusions

A feasibility study was successfully performed to demonstrate that printed bodies of FePrCuB exhibiting hard magnetic properties can be realized using additive manufacturing technology. For FePrCuB it is known from literature that permanent magnet properties can be achieved from the as-cast state by annealing without the need for subsequent powder metallurgical processing or rapid quenching. For the investigations, a pre-alloy of composition Fe73.8-Pr20.5-Cu2.0-B3.7 (at%) as well as powders and L-PBF printed parts manufactured from this were produced and characterized. The study was performed in direct comparison with conventionally fabricated as-cast and heat-treated FePrCuB of the same composition (BMC magnet). For additive manufacturing a special inert gas process chamber for laser powder bed fusion was used to safely handle the FePrCuB powders, which are extremely sensitive to oxidation. After traditional two-step annealing (step 1: homogenization at *T* = 1000 °C for 5 h followed by slow cooling to RT; step 2: annealing at 500 °C for 3 h followed by slow cooling to RT) known from as-cast magnets, the printed parts showed a coercivity of *µ*_0_*H*_c_ = 0.67 T, a remanence of *J*_r_ = 0.67 T and a maximum energy density of (*BH*)_max_ = 69.8 kJ/m^3^ at room temperature, respectively. Whereas the heat-treated BMC magnets were easy-plane permanent magnets, the printed and heat-treated L-PBF magnets were characterized by a distinct, predominantly directional microstructure that originated from the additive manufacturing process and was further refined during heat treatment. Due to the higher degree of texturing, the L-PBF magnet showed a 26% higher remanence compared to the reference BMC magnet. This corresponds to a texture grade of approximately *J*_r_/*J*_s_ = 0.67 for the L-PBF magnet.

Among the magnet materials which already have the best prerequisites in terms of their structure for permanent magnet properties (without the need for subsequent powder metallurgical processing or rapid quenching), FePrCuB is particularly promising. This is due to its relatively high saturation polarization of the hard magnetic 14:2:1 phase. Therefore, the partial texture obtained thus far shows potential in efforts to further improve the texture (and magnetic properties) of the printed parts by optimizing the processing parameters and annealing conditions. If a degree of texturing of 80% (90%) was achieved for Fe-Pr-Cu-B, the maximum energy density would be about 100 kJ/m^3^ (120 kJ/m^3^).

## Figures and Tables

**Table 1:** Overview of FePrCuB sample nomenclature, production method and sample state. Sample nomenclature is chosen according to the production route. Cast magnet materials are labeled BMC for book-mold casting; samples produced by laser powder bed fusion are labeled L-PBF for selective laser melting. Annealed samples are denoted with an additional “-a”.

Sample Name	Production Method	Annealing
BMC	BMC	-
BMC-a	BMC	1000 °C, 5 h; 500 °C, 3 h
L-PBF	L-PBF (200 W, 400 mm/s)	-
L-PBF-a	L-PBF (200 W, 400 mm/s)	1000 °C, 5 h; 500 °C, 3 h

**Table 2:** Overview of the magnetic properties coercivity *µ*_0_*H*_C_, remanence *J*_r_ and maximum energy density (*BH*)_max_ of *BMC-a* and *L-PBF-a* measured in three axes. Additionally, the magnetic properties of the as-built/as-cast state are listed.

Sample Name		*µ*_0_*H*_c_ (T)	*J*_r_ (T)	(*BH*)_max_ (kJ/m^3^)
L-PBF	field along *x* axis	0.09	0.25	4.0
BMC	field along *x* axis	0.03	0.10	0.4
L-PBF-a	field along *x* axis	0.67	0.67	69.8
	field along *y* axis	0.79	0.45	31.2
	field along *z* axis	0.75	0.26	10.4
BMC-a	field along *x* axis	1.20	0.53	42.9
	field along *y* axis	1.20	0.49	36.2
	field along *z* axis	1.22	0.32	15.3

**Table 3:** Chemical compositions (at%) of occurring phases in additively manufactured (*L-PBF/L-PBF-a*) and book-mold-cast (*BMC/BMC-a*) samples. Phases are deduced from EDX analysis.

Phase/Sample	L-PBF	L-PBF-a	BMC	BMC-a
Fe_14_Pr_2_B	Fe83.7-Pr15.3-Cu1.0	Fe82.6-Pr13.5-Cu0.3	Fe86.6-Pr13.3-Cu0.1	Fe86.8-Pr13.1-Cu0.1
Fe_17_Pr_2_	----	Fe87.9-Pr11.5-Cu0.6	----	Fe88.2-Pr11.4-Cu0.4
Fe_13_Pr_6_Cu_1_	----	Fe64.5-Pr31.3-Cu4.4	----	Fe65.8-Pr30.0-Cu4.2
α-Fe	Fe95.1-Pr4.9	----	Fe98.6-Pr1.3-Cu0.1	----
Pr-rich	Fe6.3-Pr90.7	Fe6.0-Pr93.6-Cu0.4	Fe2.6-Pr96.9-Cu0.5	Fe3.3-Pr96.1-Cu0.6
PrCu-rich	Fe18.7-Pr62.3-Cu19.0	----	Fe15.3-Pr69.8-Cu14.9	----
Pr/PrCu eutectic	----	Fe3.1Pr56.0-Cu40.9	----	Fe4.4Pr65.0-Cu30.6
total composition	Fe77.6-Pr20.6-Cu1.8	Fe77.3-Pr20.8-Cu1.9	Fe77.8-Pr20.5-Cu1.7	Fe77.4-Pr20.6-Cu2.0

## Data Availability

Data sharing not applicable.
